# lncRNAs: novel players in intervertebral disc degeneration and osteoarthritis

**DOI:** 10.1111/cpr.12313

**Published:** 2016-11-09

**Authors:** Wen‐Kang Chen, Xiao‐Hua Yu, Wei Yang, Cheng Wang, Wen‐Si He, Yi‐Guo Yan, Jian Zhang, Wen‐Jun Wang

**Affiliations:** ^1^ Department of Spine Surgery the First Affiliated Hospital University of South China Hengyang Hunan China; ^2^ Medical Research Center University of South China Hengyang Hunan China; ^3^ Department of Hand and Micro‐surgery the First Affiliated Hospital University of South China Hengyang Hunan China

**Keywords:** lncRNAs, IDD, OA, ECM, miRNAs

## Abstract

The term long non‐coding RNA (lncRNA) refers to a group of RNAs with length more than 200 nucleotides, limited protein‐coding potential, and having widespread biological functions, including regulation of transcriptional patterns and protein activity, formation of endogenous small interfering RNAs (siRNAs) and natural microRNA (miRNA) sponges. Intervertebral disc degeneration (IDD) and osteoarthritis (OA) are the most common chronic, prevalent and age‐related degenerative musculoskeletal disorders. Numbers of lncRNAs are differentially expressed in human degenerative nucleus pulposus tissue and OA cartilage. Moreover, some lncRNAs have been shown to be involved in multiple pathological processes during OA, including extracellular matrix (ECM) degradation, inflammatory responses, apoptosis and angiogenesis. In this review, we summarize current knowledge concerning lncRNAs, from their biogenesis, classification and biological functions to molecular mechanisms and therapeutic potential in IDD and OA.

**Abbreviations**
IDD: intervertebral disc degeneration
OA: osteoarthritis
ncRNAs: non‐coding RNAs
snRNAs: small nuclear RNAs
snoRNAs: small nucleolar RNAs
gRNAs: guide RNAs
miRNAs: microRNAs
siRNAs: small‐interfering RNAs
piRNAs: Piwi‐interacting RNAs
lncRNAs: long non‐coding RNAs
ORF: open reading frame
Pol II: polymerase II
dsRNAs: double‐stranded RNAs
endo‐siRNAs: endogenous siRNAs
ceRNAs: competing endogenous RNAs
NP: nucleus pulposus
AF: annulus fibrosus
CEPs: cartilaginous end plates
Col II: type II collagen
ECM: extracellular matrix
Col I: type I collagen
FAF1: Fas‐associated factor 1
SPHK1: sphingosine kinase 1
MMPs: matrix metalloproteinases
ADAMTSs: a disintegrin and metalloprotease with thrombospondin motifs
lncRNA‐CIR: cartilage injury‐related lncRNA
HOTAIR: Hox transcript antisense intergenic RNA
IL: interleukin
IGF2: insulin‐like growth factor 2
VEGF: vascular endothelial growth factor
MEG3: maternally expressed gene 3
RNAi: RNA interference
ASOs: antisense oligonucleotides
MALAT1: metastasis‐associated lung adenocarcinoma transcript 1
LNA: locked nucleic acid
ZFNs : zinc finger nucleases

## 1 Introduction

Both intervertebral disc degeneration (IDD) and osteoarthritis (OA) are the most common chronic, prevalent and age‐related degenerative musculoskeletal disorders, leading to an enormous socioeconomic burden worldwide. IDD and OA are two major causes of disability and chronic pain, and their incidence has been increasing not only among older persons but also within younger adults in the past decades. It is estimated that approximately 80% adults will suffer chronic low back pain caused by IDD during their lifetime.^1^ Over 50% of patients with symptomatic OA are younger than 65 years old.^2^ Current therapeutic options for IDD and OA are aimed at pain reduction and symptom control rather than disease modification.^3^, ^4^ Thus, a greater understanding of their pathology is important to optimize therapeutic strategies and develop novel therapeutic drugs.

Only 1% of human genomes are known to be involved in protein translation. However, 70%–90% ones are not associated with protein‐coding process, and these transcription units are called non‐coding RNAs (ncRNAs).^5^, ^6^ NcRNAs are composed of housekeeping ncRNAs and regulatory ncRNAs. Housekeeping ncRNAs include rRNA, tRNA, small nuclear RNAs (snRNAs), small nucleolar RNAs (snoRNAs), guide RNAs (gRNAs) and telomerase RNA. Regulatory ncRNAs are subdivided into two groups based on the length of their transcripts. Small ncRNAs, with less than 200 nucleotides, comprise several distinct types of ncRNAs, such as microRNAs (miRNAs), small‐interfering RNAs (siRNAs) and Piwi‐interacting RNAs (piRNAs).^7^ Long non‐coding RNAs (lncRNAs), earliest identified from cDNA in 1991, are defined as RNA>200 nucleotides in length without an open reading frame (ORF).^8^, ^9^ Current GENCODE annotation for humans (v25) lists over 15767 lncRNAs. Like miRNAs, lncRNAs are also thought to be major contributors to normal cellular physiological processes and their expression patterns are tissue‐ and cell‐specific.^10^ Although studies on lcnRNAs are still in their infancy, they have emerged as critical players in the onset and development of OA and IDD.^11^, ^12^ In this review, we overview the biogenesis, classification and functions of lncRNAs, and focus on their emerging pathological implications and therapeutic potential in IDD and OA.

## 2 Biogenesis and classification of lncRNAs

The vast majority of lncRNAs are produced by the same transcriptional machinery as are other mRNAs, as evidenced by RNA polymerase II (Pol II) occupancy and histone modifications related to transcription initiation and elongation.^13^ These lncRNAs possess a 5′ terminal methylguanosine cap and are usually spliced and polyadenylated.^14^ In addition, alternate pathways have been found to promote the generation of some lncRNAs.^15^ These pathways include a poorly characterized contingent of non‐polyadenylated lncRNAs likely expressed from RNA polymerase III promoter,^10^, ^16^ and lncRNAs that are excised during splicing and snoRNA biogenesis.^17^

**Figure 1:**
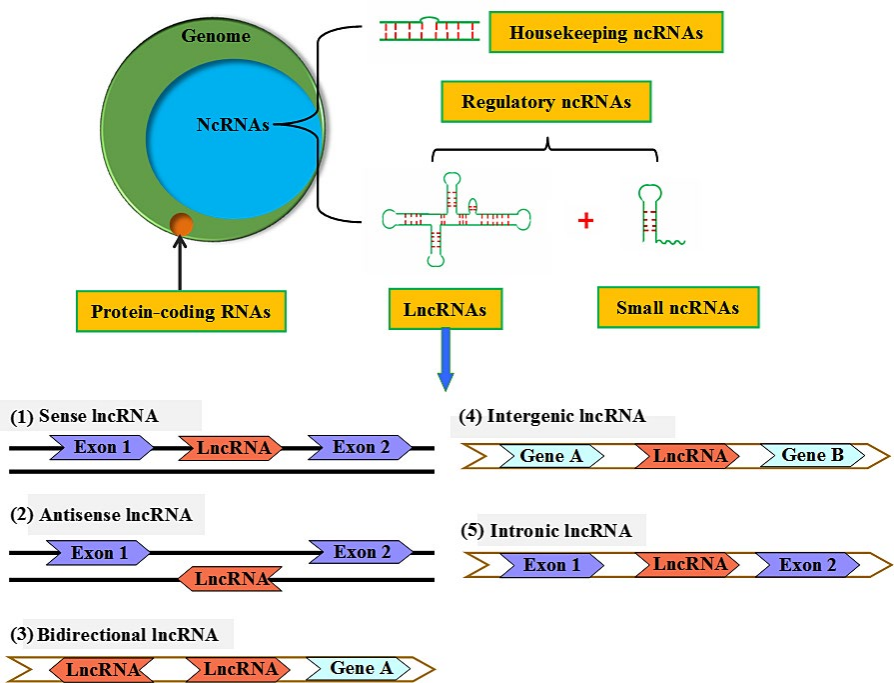
A schematic presentation of ncRNA categories. NcRNAs contain a much larger portion of the human genome than protein‐coding RNA, which comprise <3% of the genome. NcRNAs are classified into housekeeping ncRNAs, and regulatory ncRNAs that are subdivided into small ncRNAs and lncRNAs based on transcript size. LncRNAs can be designated as sense, antisense, bidirectional, intergenic or intronic according to their genomic location relative to that of nearby protein‐coding genes

Based on their locations and characteristics, lncRNAs can be classified into five subgroups (Figure 1): (1) sense (when it overlaps with one or more exons of another transcript on the same strand), (2) antisense (when it overlaps with one or more exons of another transcript on the opposite strand), (3) bidirectional (when its transcription and a neighbouring coding transcript on the opposite strand is initiated in close genomic proximity), (4) intergenic (when it lies within the genomic interval between two genes) and (5) intronic (when it transcribes from an intron of a second transcript).^18^ However, given the fact that one type of lncRNAs shares with the characteristics of other types of ones, this categorization still remains controversial.^19^ A more accurate categorization of lncRNAs is required to investigate their functions.

## 3 Biological functions of lncRNAs

**Figure 2:**
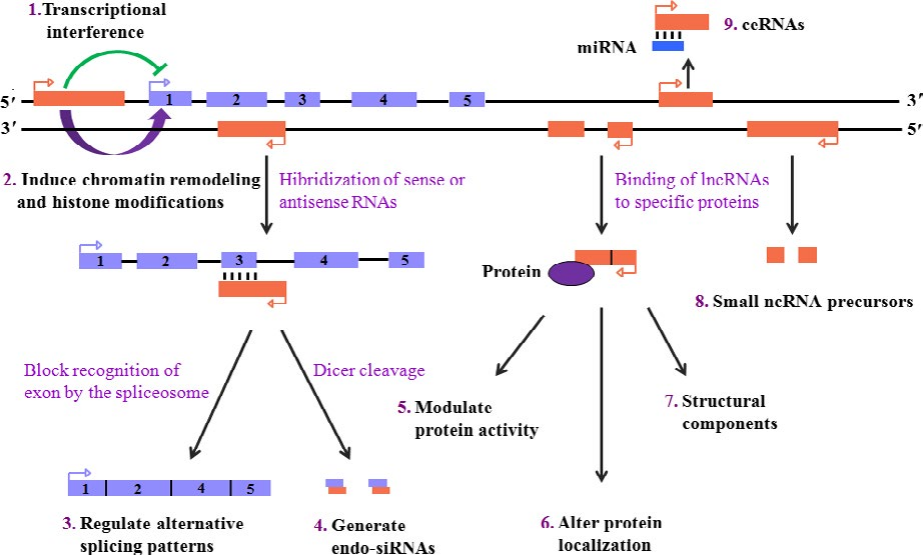
Functional paradigms of lncRNAs. LncRNAs can (1) interfere with downstream gene transcription by suppressing RNA polymerase II recruitment or (2) promote downstream gene expression *via* inducing chromatin remodelling and histone modifications. (3) An antisense lncRNA hybridizes with the complementary transcript and then disturbs recognition of the splice sites by the spliceosome for forming an alternatively spliced transcript. (4) The hybridization is also capable of producing endo‐siRNAs by Dicer cleavage. Binding of lncRNAs to specific protein partners can (5) modulate protein activity, (6) serve as structural components or (7) alter protein localization. LncRNAs act as (8) the precursors of small ncRNAs or (9) ceRNAs

As may be expected from their structural diversity, a wide range of biological functions have been described for lncRNAs. These functions primarily include gene transcription, epigenomic regulation, translation of protein‐coding genes, RNA turnover, chromatin organization and genome defence.^20^, ^21^ It is worth noting that most of the functions described to date are implicated in regulation of gene expression, both of protein‐coding genes and other non‐coding RNAs, through a multitude of mechanisms of action as presented in Figure 2.^22^ (1) Transcript of a lncRNA across the promoter region of a downstream protein‐coding gene is able to directly interfere with RNA polymerase II recruitment, thereby inhibiting the expression of protein‐coding gene.^23^ (2) LncRNAs recruit chromatin remodelers to facilitate histone modifications at specific gene loci, which can activate the transcription of target genes.^24^ (3) An antisense lncRNA can hybridize to overlap with a sense transcript, which blocks recognition of the splice sites by the spliceosome, thus leading to an alternatively spliced transcript.^25^ (4) Alternatively, lncRNAs hybridize with the sense or antisense transcripts to form double‐stranded RNAs (dsRNAs) that are subsequently cleaved by Dicer to produce endogenous siRNAs (endo‐siRNAs).^26^ (5) LncRNAs interact with specific protein partners and then regulate their activity.^27^ (6) Some lncRNAs can bind to the proteins, which forms the RNA‐protein complexes for maintaining protein stability.^28^ (7) In addition to modulating protein activity and serving as structural components, binding of lncRNAs to the proteins can affect their subcellular localization.^29^ (8) A number of lncRNAs can be post‐transcriptionally processed to generate small ncRNAs, such as miRNAs and piRNAs.^30^ (9) LncRNAs act as competing endogenous RNAs (ceRNAs) or natural miRNA sponges. These ceRNAs communicate with, and co‐regulate, each other by competing to bind to shared miRNAs, thereby titrating miRNA availability.^31^, ^32^, ^33^, ^34^ Understanding of the crosstalk between lncRNAs and miRNAs may provide novel insight into gene regulatory networks and have significant implications in human development and disease. Collectively, lncRNAs play key roles in a majority of cell molecular functions, from various aspects of transcription to translation. Although only a very small portion of known lncRNAs have been thoroughly characterized to date, future work will likely identify many more transcripts that fit into these and other functional paradigms.

## 4 Role of lncRNAs in IDD

The intervertebral disc (IVD) is a viscoelastic weight‐bearing “cushion” and plays a crucial role in the maintaining flexibility and stability of the spine. It consists of three morphologically distinct regions: nucleus pulposus (NP), annulus fibrosus (AF) and cartilaginous end plates (CEPs). Its central region is the NP, which is surrounded by the AF laterally and CEPs inferiorly and superiorly. NP is a gelatinous matrix that is rich in type II collagen (Col II) and proteoglycans, especially aggrecan.^35^ AF is a thick, dense structure, and it is divided into the outer and inner annulus. The extracellular matrix (ECM) in the outer annulus is dominated by type I collagen (Col I) and also contains relatively low amounts of proteoglycans; however, the inner annulus is made up of both Col I and Col II, with a higher proteoglycan content. It is worth noting that the IVD is the largest avascular structure in the body, with nerve endings only reaching the inner annulus.^36^ Because of these structural features, degeneration is apt to occur within the IVD.

**Figure 3:**
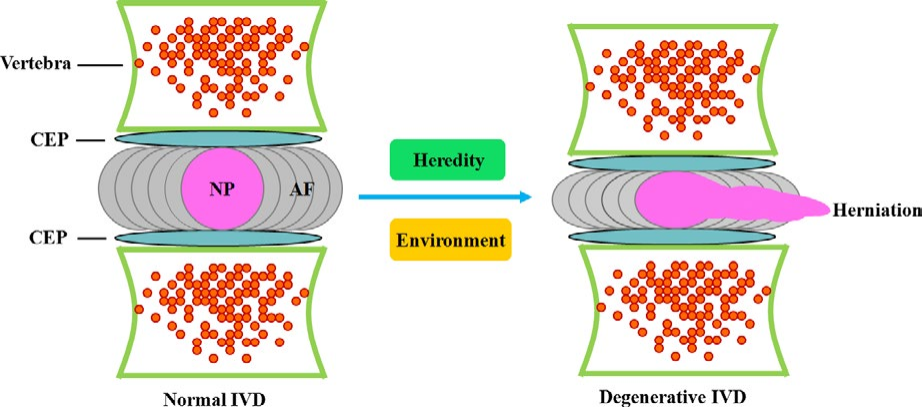
Schematic diagram of the conversion of a normal IVD to a degenerated IVD. The IVD is made up of three various regions: NP, AF and CEPs. Under the interaction between environment and heredity, a normal IVD may undergo degeneration, ultimately leading to NP herniation and low back pain

IDD is the most common cause of chronic low back pain, a public healthy question severely affecting quality of life. It constitutes the pathological foundation of most musculoskeletal disorders of the spine, including spinal stenosis, structural instability, disc herniation, radiculopathy and myelopathy.^1^ The aetiology of IDD is currently attributed to the interaction between environmental and genetic factors (Figure 3). Notably, environmental factors such as vibration, mechanical loading, physical activities and tobacco smoking are responsible for only a small portion of IDD.^37^, ^38^, ^39^ In contrast, genetic heredity is the predominant risk factor for degenerative disc disease and is estimated to cause over 70% of cases.^40^, ^41^ Although the pathogenesis of IDD is not completely understood, it is well established that progressive loss of ECM, cell proliferation, inflammatory response, angiogenesis, apoptosis, autophagy, and oxidative stress play critical roles in the occurrence and development of disc degeneration.^42^, ^43^, ^44^, ^45^, ^46^, ^47^

Like the well‐known short non‐coding RNAs such as miRNAs,^1^ lncRNAs are also involved in disc degeneration. Recently, Wan et al. detected the expression of lncRNAs in human degenerative and normal NP specimens by the lncRNA‐mRNA microarray.^48^ Among the 33045 lncRNAs and 30215 protein‐coding transcripts analysed, 116 lncRNAs (67 upregulation and 49 downregulation) and 260 mRNAs are highly differentially expressed with an absolute fold change greater than 10, and 1052 lncRNAs and 1314 mRNAs are differentially expressed in the same direction in at least four of the five degenerative samples with fold change greater than 2.^48^ The top 10 upregulated lncRNAs are ENST00000461676, ENST00000514459, ENST00000421546, AK023939, G43223, ENST00000446763, ENST00000432925, uc003mjv.3, U94385 and chr8:87319334‐87331334+, and the top 10 downregulated ones are NR_003716, nc‐HOXA13‐96‐, ENST00000451959, AK096112, nc‐HOXD1‐48+, BG897081, uc003tjk.2, NR_027154, BX646285 and AK125976.^48^ FAF1 is a multi‐domain protein as a member of the Fas death‐inducing signalling complex.^49^ Overexpression of FAF1 is known to potentiate Fas‐mediated apoptosis and suppress the degradation of ubiquitinated proteins, leading to a significant increase of cell death.^50^, ^51^ These authors also found that in addition to upregulation of the nearby enhancer‐like lncRNA, RP11‐296A18.3, FAF1 is significantly increased in degenerative discs compared with the normal discs.^48^ It has been reported that enhancer‐like lncRNAs are able to activate the proximal promoter and then stimulate transcription of their nearby coding genes.^52^ Conversely, knockout of enhancer‐like lncRNAs dramatically inhibit the expression of their neighbouring protein‐coding genes.^53^ Thus, it is likely that upregulated RP11‐296A18.3 induces overexpression of its nearby gene *FAF1*, which eventually facilitates excessive apoptosis of IVD cells. Further studies are required to test this possibility.

In a later study, a same lncRNA‐mRNA microarray technique was used to analyse the levels of lncRNAs in human degenerative and normal NP tissues.^54^ A total of 135 significantly up‐ and 170 downregulated lncRNAs as well as 2133 significantly up‐ and 1098 downregulated mRNAs are identified.^54^ Eight highly expressed lncRNAs (LINC00917, CTD‐2246P4.1, CTC‐523E23.5, RP4‐639J15.1, RP11‐363G2.4, AC005082.12, MIR132 and RP11‐38F22.1) are observed in the patterns of lncRNA‐mRNA weighted coexpression network.^54^ Moreover, sphingosine kinase 1 (SPHK1), the common interacting gene of LINC00917 and CTD‐2246P4.1, is also overexpressed in IDD tissues and is significantly enriched in positive regulation of cell migration.^54^ SPHK1 has been reported to promote endothelial cell migration and subsequent generation of neovascularization,^55^ a pathological process involving IDD. It has been recently suggested that dysregulation of lncRNAs can affect the expression of their neighbouring genes.^52^ Overexpression of SPHK1 may be caused by the upregulated LINC00917 and/or CTD‐2246P4.1, thereby leading to angiogenesis within the disc. Therefore, LINC00917 and CTD‐2246P4.1 may play a key role in the development of IDD *via* interacting with SPHK1. However, this speculation needs to be further validated by experimental evidence, and whether other differentially expressed lncRNAs are implicated in IDD is yet to be investigated.

## 5 Role of lncRNAs in OA

**Figure 4:**
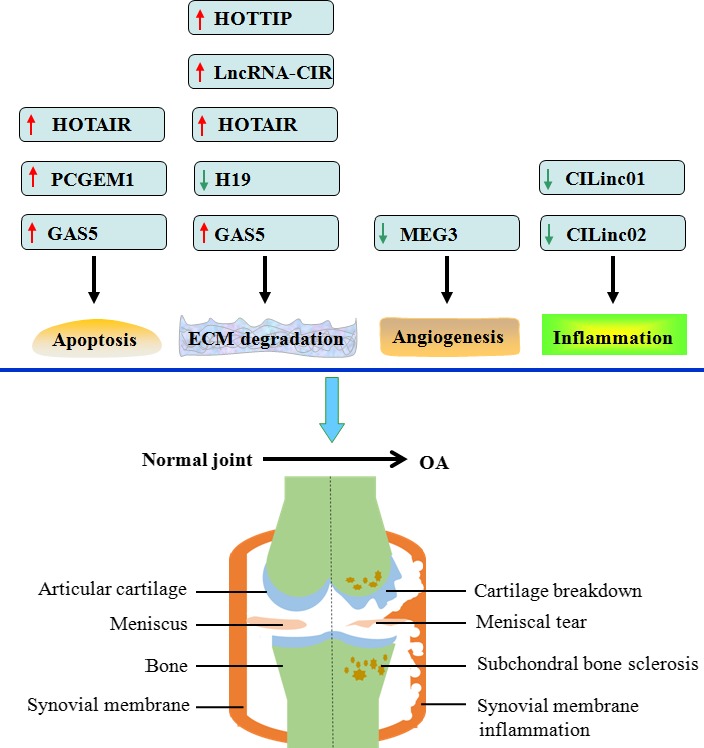
Experimentally proved lncRNAs involving OA. Apoptosis, ECM degradation, angiogenesis and inflammatory response are known to promote the development of OA. HOTAIR and GAS5 can induce chondrocyte cell apoptosis, and PCGEM1 increases the apoptotic rate of synoviocytes. HOTTIP, lncRNA‐CIR, HOTAIR and GAS5 stimulate but H19 inhibits cartilage ECM degradation. MEG3 antagonizes angiogenesis within the articular cartilage. Additionally, both CILinc01 and CILinc02 suppress inflammatory response. Red arrows indicate upregulation, and green arrows represent downregulation

OA, also called osteoarthrosis deformans, is the most common form of arthritis worldwide. As a highly heterogeneous disease, it affects all synovial joints, including the hand, knee, hip and spine. The major pathological features of OA are characterized by the progressive degradation of the articular cartilage along with secondary bone remodelling and episodic synovitis.^56^ There has been a large body of evidence supporting that ageing is the most important aetiological risk factor.^57^, ^58^ Other factors such as obesity, genetics and acute destabilising joint injuries also contribute to the development and progression of OA.^58^, ^59^, ^60^, ^61^, ^62^ Although the underlying molecular mechanisms are not fully understood, it is generally known that ECM disruption,^63^, ^64^ inflammatory response,^65^, ^66^ apoptosis^67^ and angiogenesis^68^ are closely involved in the occurrence and development of OA. Expectedly, numerous lncRNAs are differently expressed in human OA cartilage tissue, among which some ones have been shown to play a role in OA by affecting the pathological processes mentioned above (Figure 4).

### 5.1 Profile of lncRNA expression in human OA cartilage tissue

Similar to IDD, dysregulation of numerous lncRNAs exists in human OA cartilage tissue. A recent study using lncRNA microarray analysis has identified 73 upregulated lncRNAs and 48 downregulated ones in OA cartilage compared with normal cartilage.^69^ Among these differentially expressed lncRNAs, upregulation of HOTAIR, growth arrest‐specific 5 (GAS5), PMS2L2, RP11‐445H22.4, H19 and CTD‐2574D22.4 is further validated by the quantitative reverse transcription polymerase chain reaction (qRT‐PCR) technique.^69^ Fu and colleagues have demonstrated that there are 3007 upregulated lncRNAs and 1707 downregulated ones in OA cartilage when compared to normal samples.^70^ Moreover, the qRT‐PCR results of six dysregulated lncRNAs (THBS2‐1, RP11‐195E11.3, RP11‐632K21.1, SUN2, RP11‐396J17.1 and AP003175.1) are consistent with the microarray data.^70^ In another study, in comparison with normal cartilage, 82 lncRNAs are significantly upregulated, but 70 are significantly downregulated in OA cartilage.^71^ Collectively, there are a number of differently expressed lncRNAs in human OA cartilage, and these lncRNAs may be involved in the pathology of OA. Further functional investigation of single lncRNA is essential to confirm the association with OA and to explore novel potential targets for therapy.

### 5.2 lncRNAs regulate ECM degradation

Disruption of the articular cartilage followed by remodelling of the underlying bone is the hallmark of OA. The articular cartilage is an exquisitely lubricated tissue located on the surface of the joint, and it is responsible for smoothing joint articulation. Cartilage is avascular and aneural and only contains one cell type, the chondrocytes. The vast majority of cartilage volume is ECM, which is predominantly composed of Col II and aggrecan.^72^ Progressive loss of Col II and aggrecan is thought to be a main pathological feature of OA.^73^ Matrix metalloproteinases (MMPs) and a disintegrin and metalloprotease with thrombospondin motifs (ADAMTSs) are primary enzymes responsible for ECM degradation.^74^ Many members of these enzymes are highly expressed in OA cartilage tissue,^75^ and deficiency of ADAMTS‐5 leads to decreased cartilage degradation scores in a murine model of surgery‐induced OA.^76^ Given the broad biological functions of lncRNAs, it is not surprising that some miRNAs are involved in regulation of MMP and ADAMTS expression within the articular cartilage.

Recently, Liu et al. identified a novel lncRNA, named cartilage injury‐related lncRNA (lncRNA‐CIR).^71^ They found that lncRNA‐CIR expression is significantly increased in both OA cartilage and OA chondrocytes.^71^ Moreover, knockdown of lncRNA‐CIR using small‐interfering RNA (siRNA) was shown to inhibit the expression of MMP‐13 and ADAMTS‐5 and then facilitate the anabolism of Col II, Col I and aggrecan in OA chondrocytes.^71^ An opposite effect is seen in response to overexpression of lncRNA‐CIR.^71^ These findings suggest that lncRNA‐CIR contributes to ECM degradation and plays an important role in promoting the development of OA. Thus, inhibition of lncRNA‐CIR may provide a novel and promising therapeutic strategy for stimulating ECM regeneration and mitigating joint degeneration.

Hox transcript antisense intergenic RNA (HOTAIR), a trans‐acting lncRNA, was introduced by Rinn et al. as a spliced and polyadenylated RNA containing 2158 nucleotides and 6 exons.^77^ This lncRNA arises from the transcription of antisense strand of *HoxC* gene, which is specifically situated between *HoxC11* and *HoxC12* on chromosome 12q13.13.^78^ It has been reported that HOTAIR expression is significantly increased in hepatocellular cancer,^79^ gastric cancer,^80^ non‐small cell lung cancer 81 and acute myeloid leukaemia.^82^ Overexpression of HOTAIR markedly promotes MMP‐9 synthesis in SiHa cells, a human cervical cancer cell line.^83^ Similarly, HOTAIR is significantly upregulated in the synovial fluid of temporomandibular joint OA patients compared with that of normal controls.^84^ Increased HOTAIR levels are also observed in the synovial fluid of temporomandibular joint OA rabbits as compared to control rabbits.^84^ Furthermore, in chondrocytes isolated from the temporomandibular joint condylar cartilage of New Zealand white rabbits, interleukin (IL)‐1β treatment dramatically enhances the expression of MMP‐1, MMP‐3 and MMP‐9, whereas these effects are reversed by HOTAIR knockdown.^84^ Taken together, these observations support the idea that HOTAIR is involved in upregulation of MMPs induced by IL‐1β and also functions as a contributor to OA.

H19 was the first lncRNA gene discovered,^85^ and is paternally imprinted. In humans, H19 maps to chromosome 11p15.5, in close proximity to the reciprocally imprinted insulin‐like growth factor 2 (IGF2) gene.^86^ It transcribes a 2.3 kb non‐coding RNA transcript by RNA polymerase II and splices to five exons. H19 is highly expressed throughout development of the embryo and foetus, and also to the large extent in the placenta, but it is shut down in the vast majority of tissues shortly after birth. In cultured human chondrocytes under chemically induced hypoxic condition, the expression of H19 and Col IIA1 (a subtype of Col II) is significantly increased, and a positive correlation exists between H19 and Col IIA1 levels.^87^ H19 is known to serve as a precursor for miR‐675.^88^ Transfection with the miR‐675 mimic also dramatically enhances Col IIA1 levels in human normal chondrocytes lacking H19.^89^ These findings suggest a protective role for H19 in regulating the balance between matrix anabolism and catabolism within the articular cartilage.

HOTTIP resides in 5′ end of the HoxA cluster and encodes a lncRNA that is known to downregulate the expression of HoxA‐13, a murine abdominal‐B‐type homeobox gene, one of the members in the Hox gene family, by directly inhibiting histone modifications.^90^, ^91^ Knockout of HoxA‐13 has been shown to cause digital reduction.^92^ Also, mis‐expression of HoxA‐13 in chick limb leads to homeotic transformation of the cartilage condensations and reduces cell death by altering cell adhesiveness in chick limb buds.^93^ In human OA chondrocytes, HOTTIP expression is significantly increased, with concurrent downregulation of HoxA‐13.^94^ Moreover, introduction of HoxA‐13 siRNA to human OA chondrocytes markedly decreases integrin‐α1 levels.^94^ Of important, overexpression of integrin‐α1 subunit contributes to chondrogenesis, and mice lacking integrin‐α1 develop cartilage degradation at a younger age and show increased MMP‐2 synthesis.^95^ Thus, HOTTIP may promote the degradation of cartilage ECM by suppressing the HoxA‐13/integrin‐α1/MMP‐2 signalling pathway, which plays an important role in the pathogenesis of OA. Nevertheless, this speculation needs to be further proved by experimental evidence, and whether HOTTIP can affect other MMP production for ECM loss in OA is yet to be investigated.

In addition to lncRNA‐CIR, HOTAIR, H19 and HOTTIP, GAS5 is associated with modulation of ECM anabolism and catabolism in chondrocytes. GAS5 was originally identified from a subtraction cDNA library and named after the finding that its expression levels increased upon growth arrest in mammalian cells.^96^ It is located at 1q25 and contains 12 exons that are alternatively spliced to produce two possible mature lncRNAs (GAS5a and GAS5b) and 11 introns. These introns can encode 10 box C/D snoRNA. In spite of its short ORF, GAS5 does not possess protein‐coding capacity and serves as a so‐called host gene for snoRNAs.^97^ In SK‐Mel‐110 cells, a human melanoma cell line, overexpression of GAS5 markedly downregulates the expression of MMP‐2, leading to reduced migration and invasion ability.^98^ Conversely, GAS5 levels are higher in human OA chondrocytes than those in normal chondrocytes, and its exogenous induction in human OA chondrocytes diminishes miR‐21 levels and then increases the expression of MMP‐2, MMP‐3, MMP‐9, MMP‐13 and ADAMTS‐4, with concomitant decrease in the Col II and aggrecan contents.^99^ This discrepancy may be attributed to the differences in the types of cells, and cultural system.

### 5.3 lncRNAs regulate the inflammatory response

Inflammation is increasingly being recognized as an important driver of OA cartilage pathology.^100^, ^101^, ^102^ A variety of proinflammatory cytokines including IL‐1β, tumour necrosis factor‐α (TNF‐α), IL‐6, IL‐15, IL‐17, IL‐18 and IL‐21 have been reported to be elevated in the synovial fluid of OA joints, with substantial infiltration of activated B cells and T lymphocytes.^103^ Moreover, cytokine stimulation of *ex vivo* cartilage tissue mimics the pathological changes observed within the OA joint.^104^ Like miRNAs, lncRNAs are also involved in the regulation of OA inflammation.^105^ For example, Pearson et al. identified two novel chondrocyte inflammation‐associated lincRNAs, named CILinc01 and CILinc02.^106^ They found that their expression is significantly decreased in both knee and hip OA cartilage compared with non‐OA cartilage.^106^ In human chondrocyte TC28 cell line, knockdown of CILinc01 or CILinc02 dramatically enhances IL‐1β‐stimulated production of IL‐6, IL‐8, TNF‐α, macrophage inflammatory protein (MIP‐1β) and granulocyte‐colony stimulating factor (G‐CSF), suggesting a protective role of these two lncRNAs in controlling inflammation‐driven cartilage degeneration in OA.

### 5.4 LncRNAs regulate chondrocyte and synoviocyte apoptosis

Apoptosis, also called type I programmed cell death, is a highly regulated pathway that involves specific sets of intracellular signals and genes. Dysregulation of apoptosis results in pathological states, such as cancer, developmental anomalies and degenerative diseases.^107^, ^108^ During apoptosis, cells show morphological characteristics including cell shrinkage, plasma membrane blebbing, chromatin condensation, DNA fragmentation and apoptotic body formation.^109^ Chondrocytes play a crucial role in maintaining articular integrity and physiology *via* the synthesis of ECM components to resist mechanical loads. It is well established that reduced chondrocyte number due to apoptosis is a critical cause leading to cartilage degeneration in the process of OA.^67^, ^110^ Interestingly, some studies have focused on the association of lncRNAs with chondrocyte apoptosis. Several lines of evidence have revealed GAS5 as an inducer of apoptosis in numerous human cancers, such as breast cancer,^111^ hepatocellular carcinoma,^112^ ovarian cancer 113 and prostate cancer.^114^ Similarly, the exogenous induction of GAS5 in normal human chondrocytes decreases miR‐21 levels and then stimulate apoptosis, leading to a severe degenerative morphology.^99^ In primary rabbit condylar chondrocytes, knockdown of HOTAIR is also found to attenuate the apoptosis rate induced by IL‐1β.^84^ Therefore, inhibition of GAS5 or HOTAIR may have a potential therapeutic benefit for OA patients through the blockage of chondrocyte apoptosis.

Synovitis is one of the most important characteristics of OA, presenting with pathological features including hyperplasia of the synovial lining and inflammatory cell infiltration.^115^ Activated synoviocytes secrete a number of proinflammatory cytokines such as IL‐1β, TNF‐α and IL‐6, which block cartilage turnover and promote the produce of MMPs and cathepsins, resulting in the destruction of bone and cartilage.^116^ In contradiction with chondrocytes, increased number of synoviocytes thus contributes to the development of OA. A recent experimental study by Kang et al. has demonstrated that prostate cancer gene expression marker 1 (PCGEM1), a lncRNA, is highly expressed in human OA synoviocytes.^117^ Moreover, exogenous overexpression of PCGEM1 markedly inhibits apoptosis and stimulates the proliferation of human OA synoviocytes by directly binding to miR‐770.^117^ Thus, reduced synoviocyte apoptosis may be one of the mechanisms by which activation of PCGEM1 promotes OA progression.

### 5.5 LncRNAs regulate the angiogenesis

Angiogenesis, defined as blood vessel outgrowth from pre‐existing vasculature, is indispensable for growth and development, the reproductive cycle and tissue repair.^118^ As articular cartilage and the inner two‐thirds of the meniscus are normally avascular, they depend on oxygen and nutrients from adjacent synoviocytes and synovial blood vessels *via* the synovial fluid.^119^, ^120^ There has been a large body of evidence supporting the involvement of angiogenesis in OA development.^121^, ^122^, ^123^ Angiogenesis is modulated by the equilibrium of proangiogenic and anti‐angiogenic factors, which is regulated by the presence of either a facilitating or inhibitory ECM environment. Pathological neovascularization in OA represents a breakdown of these normal homeostatic mechanisms. Although the precise molecular pathways that regulate angiogenesis in the osteoarthritic joint are not still fully understood, proangiogenic factors produced within the osteoarthritic joint include prostaglandins, nitric oxide, regulatory peptides, cytokines, chemokines and growth factors, particularly vascular endothelial growth factor (VEGF). Anti‐angiogenic factors contain protease inhibitors, matrix fragments and factors involved in the regression of inflammation.^123^, ^124^

Maternally expressed gene 3 (MEG3) is an imprinted gene that belongs to the imprinted delta‐like 1 homologue (DLK1)‐MEG3 locus located at chromosome 14q32.3 in humans. Its mouse orthologue, Meg3, also known as gene trap locus 2 (Gtl2), is localized to distal chromosome 12. The MEG3 gene encodes a lncRNA, which is expressed in many normal tissues. It has already been reported that VEGF expression and cortical microvessel formation are significantly increased in the brains of mouse Meg3‐null embryos.^125^, ^126^ In agreement, downregulated MEG3 and upregulated VEGF are observed in articular cartilage samples from OA patients compared with normal controls.^127^ Moreover, MEG3 levels are inversely correlated with VEGF levels.^127^ Recently, Zhou et al. have demonstrated that MEG3 stimulates p53 transcription by binding to the transcription factor Sp1 sites on the VEGF promoter.^128^ Thus, it is possible that downregulation of MEG3 promotes VEGF transcription *via* decreasing p53 activity, leading to angiogenesis in OA. However, further research is required to test this possibility. Restoration of MEG3 expression may represent a novel and promising therapeutic strategy to suppress osteoarthritic angiogenesis.

## 6 Possible therapeutic approaches for targeting lncRNAs in IDD and OA

The onset and development of IDD and OA has been regarded as a multifactorial and complex process. Currently, there is still a lack of effectively biological treatment in IDD and OA patients, highlighting the need to search for novel therapeutic approaches. As mentioned above, many lncRNAs are differently expressed in human degenerative IVD tissue and OA cartilage tissue and some lncRNAs have been shown to involve multiple pathological processes of OA development, suggesting lncRNAs as novel contributors to both diseases. Thus, lncRNA targeting therapy is expected to open a new hope for the management of IDD and OA.

### 6.1 Silencing of lncRNAs

RNA interference (RNAi) is a process through which double‐stranded RNA induces the activation of cellular pathways, leading to potent and selective silencing of genes with homology to the double strand.^129^ Induction of RNAi through administration of siRNAs has been successfully used in treatment of hepatitis, viral infections and cancers.^130^ Similar to other genes, lncRNAs are also able to be silenced by using specific siRNAs. For example, siRNA against HOTAIR appears to protect against OA development.^84^ However, several obstacles need to be overcome in the application of RNAi to knockdown a lncRNA of interest for therapeutic purposes: (1) siRNAs often do not only silence their specific target genes but also interfere with the expression of other genes (off‐target effects).^131^ (2) The efficiency of a siRNA is not predictable, so that finding a good – efficient and specific – siRNA can be time and cost‐intensive. (3) Some transcripts can be hard to target because of their strong secondary structure, incorporation into large protein complexes or their intracellular localization. (4) The siRNA‐mediated knockdown is not permanent, making it unsuitable for long‐term treatment.

### 6.2 Antisense oligonucleotides

Antisense oligonucleotides (ASOs) are single‐stranded nucleotides or nucleotide analogues, which predominantly act in the nucleus by selectively cleaving pre‐mRNAs having complementary sites *via* an RNase H‐dependent mechanism.^132^ Although ASOs can also act by translation arrest, they are currently primarily used as ‘GapmeRs’, having a central region that supports RNase H activity flanked by chemically modified ends that enhance affinity and decrease susceptibility to nucleases.^133^ Like siRNAs, ASOs are a viable approach to target lncRNAs for therapeutic purposes. In recent years, targeting lncRNAs with ASO technology has been applied in cancer therapy.^134^ For example, blockade of lncRNA MALAT1 (metastasis‐associated lung adenocarcinoma transcript 1) using ASO has been shown to inhibit metastasis formation after lung cancer implantation.^135^ It is anticipated that lncRNA ASOs could be potential candidates for treating IDD and OA. However, effective delivery of ASOs to their intracellular sites of action remains a major challenge.

### 6.3 Locked nucleic acid GapmeRs

Locked nucleic acid (LNA) GapmeRs are single‐stranded oligonucleotides, which comprise a DNA stretch flanked by LNA nucleotides and, similar to ASOs, form base pairs with targeting lncRNAs to induce their degradation *via* a RNAse H‐dependent mechanism.^136^ A recent study by Michalik et al. has demonstrated that silencing of MALAT1 by GapmeRs markedly attenuates blood flow recovery and capillary density after hindlimb ischaemia.^137^ Hence, this approach to target lncRNAs may also have potential therapeutic application in the treatment of IDD and OA.

### 6.4 Small molecule inhibitors

Small molecule inhibitors are another therapeutic strategy. These inhibitors are designed to suppress lncRNA expression or to hide the binding sites for lncRNAs and thereby antagonize the interaction with specific partners. It has been reported that bromodomain‐containing 4 (BRD4), a small molecule inhibitor, can downregulate HOTAIR expression and then inhibit glioblastoma tumour growth.^138^ Another small molecule inhibitor that blocks the binding of HOTAIR to its partners is also found to protect against tumour cell proliferation, invasion and metastasis.^139^ Given the fact that HOTAIR plays an important role in promoting OA progression,^84^ in vivo delivery of small molecule inhibitors for HOTAIR may have a potential therapeutic benefit for OA patients.

### 6.5 Zinc finger nucleases

Zinc finger nucleases (ZFNs) are genetically engineered proteins that contain a DNA‐binding domain composed of at least three Cys_2_His_2_ zinc fingers and a non‐specific DNA cleavage domain derived from the restriction endonuclease FokI.^140^ The zinc finger domains can be engineered to target a specific nucleotide sequence. The fused nuclease domain creates a DNA double‐strand break at this specific site after dimerization, thus conferring a knockout‐like effect on gene functions. Recently, ZFNs have been successfully employed to downregulate MALAT1 expression, with a 1000‐fold reduction.^141^ Thus, this approach is far superior to RNAi technique so as to knockdown a lncRNA.

## 7 Conclusions and future directions

Despite the investigations of lcnRNAs remaining in their infancy, they have been suggested as new contributors to IDD and OA. This provides novel insight into the pathogenesis of IDD and OA. With continued efforts, some dysregulated lncRNAs may be used as valuable diagnostic biomarkers and therapeutic targets. However, many challenges are still ahead. Thousands of lncRNAs have been shown to be differentially expressed in IDD and OA, whereas there is still a lack of the effects of single lncRNA on IDD and only nine lncRNAs are proved to involve OA to date. Thus, it is important to elucidate the role of more other lncRNAs in IDD and OA development. LncRNAs are known to act as natural miRNA sponges, and there has been a large body of evidence supporting the involvement of miRNAs in IDD 1 and OA.^142^, ^143^, ^144^ Future studies should also focus on the novel crosstalk between lncRNAs and miRNAs during IDD and OA. Although in vitro studies may provide promising results with regard to lncRNAs involving both diseases, in vivo validation will be necessary. As discussed above, several approaches to target lncRNAs for therapeutic purposes can be considered once key disease‐relevant contributions of these genes have been identified. A major challenge of all of these approaches is to accomplish target‐specific delivery. Recently, several novel delivery strategies have been developed to reduce off‐target effects, especially nanoparticles that are characteristic by improved stability, extremely small size, biocompatibility and self‐assembly.^145^, ^146^ Administration of HOTAIR siRNA by using magnetic nanoparticles has been shown to effectively inhibit the proliferation, invasion and in vivo tumourigenicity of human glioma stem cells.^147^ The use of nanoparticles as effective delivery vehicles for lncRNAs is thus highly attractive and deserves further studies. As the pace of research in lncRNAs progresses, addressing these issues will provide opportunities for the development of novel therapeutic strategies based on targeting lncRNAs for IDD and OA.

## Conflict of interest

The authors have declared no conflict of interest.

## References

[1] Wang C , Wang WJ , Yan YG , et al. MicroRNAs: New players in intervertebral disc degeneration. Clin Chim Acta. 2015;450:333–341. 26368266 10.1016/j.cca.2015.09.011

[2] Deshpande BR , Katz JN , Solomon DH , et al. The number of persons with symptomatic knee osteoarthritis in the United States: Impact of race/ethnicity, age, sex, and obesity. Arthritis Care Res (Hoboken). 2016; doi: 10.1002/acr.22897. [Epub ahead of print]. PMC5319385 27014966

[3] Veronesi F , Della Bella E , Cepollaro S , Brogini S , Martini L , Fini M . Novel therapeutic targets in osteoarthritis: Narrative review on knock‐out genes involved in disease development in mouse animal models. Cytotherapy. 2016;18:593–612. 27059198 10.1016/j.jcyt.2016.02.001

[4] Wang WJ , Yu XH , Wang C , et al. MMPs and ADAMTSs in intervertebral disc degeneration. Clin Chim Acta. 2015;448:238–246. 26162271 10.1016/j.cca.2015.06.023

[5] Batista PJ , Chang HY . Long noncoding RNAs: cellular address codes in development and disease. Cell. 2013;152:1298–1307. 23498938 10.1016/j.cell.2013.02.012 PMC3651923

[6] Lee JT . Epigenetic regulation by long noncoding RNAs. Science. 2012;338:1435–1439. 23239728 10.1126/science.1231776

[7] Maxmen A . RNA: The genome's rising stars. Nature. 2013;496:127–129. 23565521 10.1038/nj7443-127a

[8] Brown CJ , Ballabio A , Rupert JL , et al. A gene from the region of the human X inactivation centre is expressed exclusively from the inactive X chromosome. Nature. 1991;349:38–44. 1985261 10.1038/349038a0

[9] Bartolomei MS , Zemel S , Tilghman SM . Parental imprinting of the mouse H19 gene. Nature. 1991;351:153–155. 1709450 10.1038/351153a0

[10] Derrien T , Johnson R , Bussotti G , et al. The GENCODE v7 catalog of human long noncoding RNAs: analysis of their gene structure, evolution, and expression. Genome Res. 2012;22:1775–1789. 22955988 10.1101/gr.132159.111 PMC3431493

[11] Dey BK , Mueller AC , Dutta A . Long non‐coding RNAs as emerging regulators of differentiation, development, and disease. Transcription. 2014;5:e944014. 25483404 10.4161/21541272.2014.944014 PMC4581346

[12] Liu X , Che L , Xie YK , et al. Noncoding RNAs in human intervertebral disc degeneration: An integrated microarray study. Genom Data. 2015;5:80–81. 26484230 10.1016/j.gdata.2015.05.027 PMC4583642

[13] Guttman M , Amit I , Garber M , et al. Chromatin signature reveals over a thousand highly conserved large non‐coding RNAs in mammals. Nature. 2009;458:223–227. 19182780 10.1038/nature07672 PMC2754849

[14] Mercer TR , Mattick JS . Structure and function of long noncoding RNAs in epigenetic regulation. Nat Struct Mol Biol. 2013;20:300–307. 23463315 10.1038/nsmb.2480

[15] Kapranov P , Cheng J , Dike S , et al. RNA maps reveal new RNA classes and a possible function for pervasive transcription. Science. 2007;316:1484–1488. 17510325 10.1126/science.1138341

[16] Kiyosawa H , Mise N , Iwase S , Hayashizaki Y , Abe K . Disclosing hidden transcripts: mouse natural sense‐antisense transcripts tend to be poly(A) negative and nuclear localized. Genome Res. 2005;15:463–474. 15781571 10.1101/gr.3155905 PMC1074361

[17] Yin QF , Yang L , Zhang Y , et al. Long noncoding RNAs with snoRNA ends. Mol Cell. 2012;48:219–230. 22959273 10.1016/j.molcel.2012.07.033

[18] Ponting CP , Oliver PL , Reik W . Evolution and functions of long noncoding RNAs. Cell. 2009;136:629–641. 19239885 10.1016/j.cell.2009.02.006

[19] Dozmorov MG , Giles CB , Koelsch KA , Wren JD . Systematic classification of non‐coding RNAs by epigenomic similarity. BMC Bioinformatics. 2013;14:S2. 10.1186/1471-2105-14-S14-S2 PMC3851203 24267974

[20] Ferraiuolo MA , Rousseau M , Miyamoto C , et al. The three‐dimensional architecture of Hox cluster silencing. Nucleic Acids Res. 2010;38:7472–7484. 20660483 10.1093/nar/gkq644 PMC2995065

[21] Parasramka MA , Maji S , Matsuda A , Yan IK , Patel T . Long non‐coding RNAs as novel targets for therapy in hepatocellular carcinoma. Pharmacol Ther. 2016;161:67–78. 27013343 10.1016/j.pharmthera.2016.03.004 PMC4851900

[22] Tekcham DS , Tiwari PK . Non‐coding RNAs as emerging molecular targets of gallbladder cancer. Gene. 2016;588:79–85. 27131889 10.1016/j.gene.2016.04.047

[23] Martianov I , Ramadass A , Serra Barros A , Chow N , Akoulitchev A . Repression of the human dihydrofolate reductase gene by a non‐coding interfering transcript. Nature. 2007;445:666–670. 17237763 10.1038/nature05519

[24] Khalil AM , Guttman M , Huarte M , et al. Many human large intergenic noncoding RNAs associate with chromatin‐modifying complexes and affect gene expression. Proc Natl Acad Sci USA. 2009;106:11667–11672. 19571010 10.1073/pnas.0904715106 PMC2704857

[25] Tripathi V , Ellis JD , Shen Z , et al. The nuclear‐retained noncoding RNA MALAT1 regulates alternative splicing by modulating SR splicing factor phosphorylation. Mol Cell. 2010;39:925–938. 20797886 10.1016/j.molcel.2010.08.011 PMC4158944

[26] Taylor DH , Chu ET , Spektor R , Soloway PD . Long non‐coding RNA regulation of reproduction and development. Mol Reprod Dev. 2015;82:932–956. 26517592 10.1002/mrd.22581 PMC4762656

[27] Feng J , Bi C , Clark BS , Mady R , Shah P , Kohtz JD . The Evf‐2 noncoding RNA is transcribed from the Dlx‐5/6 ultraconserved region and functions as a Dlx‐2 transcriptional coactivator. Genes Dev. 2006;20:1470–1484. 16705037 10.1101/gad.1416106 PMC1475760

[28] Yao Y , Li J , Wang L . Large intervening non‐coding RNA HOTAIR is an indicator of poor prognosis and a therapeutic target in human cancers. Int J Mol Sci. 2014;15:18985–18999. 25334066 10.3390/ijms151018985 PMC4227256

[29] Prasanth KV , Prasanth SG , Xuan Z , et al. Regulating gene expression through RNA nuclear retention. Cell. 2005;123:249–263. 16239143 10.1016/j.cell.2005.08.033

[30] Rashid F , Shah A , Shan G . Long Non‐coding RNAs in the Cytoplasm. Genomics Proteomics Bioinformatics. 2016;14:73–80. 27163185 10.1016/j.gpb.2016.03.005 PMC4880952

[31] Tay Y , Rinn J , Pandolfi PP . The multilayered complexity of ceRNA crosstalk and competition. Nature. 2014;505:344–352. 24429633 10.1038/nature12986 PMC4113481

[32] Yang C , Wu D , Gao L , et al. Competing endogenous RNA networks in human cancer: hypothesis, validation, and perspectives. Oncotarget. 2016;7:13479–13490. 26872371 10.18632/oncotarget.7266 PMC4924655

[33] Hu Y , Tian H , Xu J , Fang JY . Roles of competing endogenous RNAs in gastric cancer. Brief Funct Genomics. 2016;15:266–273. 26404556 10.1093/bfgp/elv036

[34] Karreth FA , Pandolfi PP . ceRNA cross‐talk in cancer: when ce‐bling rivalries go awry. Cancer Discov. 2013;3:1113–1121. 24072616 10.1158/2159-8290.CD-13-0202 PMC3801300

[35] Woods BI , Vo N , Sowa G , Kang JD . Gene therapy for intervertebral disk degeneration. Orthop Clin North Am 2011;42:563–574. 21944592 10.1016/j.ocl.2011.07.002

[36] Boubriak OA , Watson N , Sivan SS , Stubbens N , Urban JP . Factors regulating viable cell density in the intervertebral disc: blood supply in relation to disc height. J Anat. 2013;222:341–348. 23311982 10.1111/joa.12022 PMC3582253

[37] Petit A , Roquelaure Y . Low back pain, intervertebral disc and occupational diseases. Int J Occup Saf Ergon. 2015;21:15–19. 26327258 10.1080/10803548.2015.1017940

[38] Paul CP , Zuiderbaan HA , Zandieh Doulabi B , et al. Simulated‐physiological loading conditions preserve biological and mechanical properties of caprine lumbar intervertebral discs in ex vivo culture. PLoS ONE. 2012;7:e33147. 22427972 10.1371/journal.pone.0033147 PMC3302815

[39] Kalb S , Martirosyan NL , Kalani MY , Broc GG , Theodore N . Genetics of the degenerated intervertebral disc. World Neurosurg. 2012;77:491–501. 22120330 10.1016/j.wneu.2011.07.014

[40] Battie MC , Videman T , Parent E . Lumbar disc degeneration: epidemiology and genetic influences. Spine (Phila Pa 1976). 2004;29:2679–2690. 15564917 10.1097/01.brs.0000146457.83240.eb

[41] Battie MC , Videman T . Lumbar disc degeneration: epidemiology and genetics. J Bone Joint Surg Am. 2006;88(Suppl 2):3–9. 16595435 10.2106/JBJS.E.01313

[42] Hwang MH , Kim KS , Yoo CM , et al. Photobiomodulation on human annulus fibrosus cells during the intervertebral disk degeneration: extracellular matrix‐modifying enzymes. Lasers Med Sci. 2016;31:767–777. 26987527 10.1007/s10103-016-1923-x

[43] Hwang MH , Kim KS , Yoo CM , et al. Low level light therapy modulates inflammatory mediators secreted by human annulus fibrosus cells during intervertebral disc degeneration in vitro. Photochem Photobiol. 2015;91:403–410. 25557915 10.1111/php.12415

[44] Binch AL , Cole AA , Breakwell LM , et al. Class 3 semaphorins expression and association with innervation and angiogenesis within the degenerate human intervertebral disc. Oncotarget. 2015;6:18338–18354. 26286962 10.18632/oncotarget.4274 PMC4621894

[45] Liu Z , Ma C , Shen J , Wang D , Hao J , Hu Z . SDF1/CXCR4 axis induces apoptosis of human degenerative nucleus pulposus cells via the NFkappaB pathway. Mol Med Rep. 2016;14:783–789. 27220474 10.3892/mmr.2016.5341 PMC4918601

[46] Zhang SJ , Yang W , Wang C , et al. Autophagy: A double‐edged sword in intervertebral disk degeneration. Clin Chim Acta. 2016;457:27–35. 27018178 10.1016/j.cca.2016.03.016

[47] Krupkova O , Handa J , Hlavna M , et al. The natural polyphenol epigallocatechin gallate protects intervertebral disc cells from oxidative stress. Oxid Med Cell Longev. 2016;2016:7031397. 27119009 10.1155/2016/7031397 PMC4826942

[48] Wan ZY , Song F , Sun Z , et al. Aberrantly expressed long noncoding RNAs in human intervertebral disc degeneration: a microarray related study. Arthritis Res Ther. 2014;16:465. 25280944 10.1186/s13075-014-0465-5 PMC4201740

[49] Song J , Park JK , Lee JJ , et al. Structure and interaction of ubiquitin‐associated domain of human Fas‐associated factor 1. Protein Sci. 2009;18:2265–2276. 19722279 10.1002/pro.237 PMC2788281

[50] Park MY , Ryu SW , Kim KD , Lim JS , Lee ZW , Kim E . Fas‐associated factor‐1 mediates chemotherapeutic‐induced apoptosis via death effector filament formation. Int J Cancer. 2005;115:412–418. 15688372 10.1002/ijc.20857

[51] Chu K , Niu X , Williams LT . A Fas‐associated protein factor, FAF1, potentiates Fas‐mediated apoptosis. Proc Natl Acad Sci USA. 1995;92:11894–11898. 8524870 10.1073/pnas.92.25.11894 PMC40509

[52] Orom UA , Derrien T , Beringer M , et al. Long noncoding RNAs with enhancer‐like function in human cells. Cell. 2010;143:46–58. 20887892 10.1016/j.cell.2010.09.001 PMC4108080

[53] Shiekhattar R . The Yin and Yang of enhancer‐like RNAs. EMBO J. 2013;32:2533–2534. 23942236 10.1038/emboj.2013.185 PMC3791368

[54] Chen Y , Ni H , Zhao Y , et al. Potential role of lncRNAs in contributing to pathogenesis of intervertebral disc degeneration based on microarray data. Med Sci Monit. 2015;21:3449–3458. 26556537 10.12659/MSM.894638 PMC4646231

[55] Limaye V , Li X , Hahn C , et al. Sphingosine kinase‐1 enhances endothelial cell survival through a PECAM‐1‐dependent activation of PI‐3K/Akt and regulation of Bcl‐2 family members. Blood. 2005;105:3169–3177. 15632208 10.1182/blood-2004-02-0452

[56] Findlay DM , Atkins GJ . Osteoblast‐chondrocyte interactions in osteoarthritis. Curr Osteoporos Rep. 2014;12:127–134. 24458429 10.1007/s11914-014-0192-5 PMC3933767

[57] Loeser RF , Collins JA , Diekman BO . Ageing and the pathogenesis of osteoarthritis. Nat Rev Rheumatol. 2016;7:412–420. 10.1038/nrrheum.2016.65 PMC4938009 27192932

[58] Greene MA , Loeser RF . Aging‐related inflammation in osteoarthritis. Osteoarthritis Cartilage. 2015;23:1966–1971. 26521742 10.1016/j.joca.2015.01.008 PMC4630808

[59] Lee S , Kim TN , Kim SH , et al. Obesity, metabolic abnormality, and knee osteoarthritis: a cross‐sectional study in Korean women. Mod Rheumatol. 2015;25:292–297. 25065916 10.3109/14397595.2014.939393

[60] Wang Q , Yan XB , Sun QQ , Hu AM , Liu HL , Yin YW . Genetic polymorphism of the estrogen receptor alpha gene and susceptibility to osteoarthritis: evidence based on 15,022 subjects. Curr Med Res Opin. 2015;31:1047–1055. 25892216 10.1185/03007995.2015.1037727

[61] Lieberthal J , Sambamurthy N , Scanzello CR . Inflammation in joint injury and post‐traumatic osteoarthritis. Osteoarthritis Cartilage. 2015;23:1825–1834. 26521728 10.1016/j.joca.2015.08.015 PMC4630675

[62] Visser AW , de Mutsert R , le Cessie S , den Heijer M , Rosendaal FR , Kloppenburg M . The relative contribution of mechanical stress and systemic processes in different types of osteoarthritis: the NEO study. Ann Rheum Dis. 2015;74:1842–1847. 24845389 10.1136/annrheumdis-2013-205012

[63] Lee AS , Ellman MB , Yan D , et al. A current review of molecular mechanisms regarding osteoarthritis and pain. Gene. 2013;527:440–447. 23830938 10.1016/j.gene.2013.05.069 PMC3745800

[64] Liu Q , Zhang X , Hu X , et al. Circular RNA Related to the Chondrocyte ECM Regulates MMP13 Expression by Functioning as a MiR‐136 ‘Sponge’ in Human Cartilage Degradation. Sci Rep. 2016;6:22572. 26931159 10.1038/srep22572 PMC4773870

[65] Maldonado M , Nam J . The role of changes in extracellular matrix of cartilage in the presence of inflammation on the pathology of osteoarthritis. Biomed Res Int. 2013;2013:284873. 24069595 10.1155/2013/284873 PMC3771246

[66] Goldring MB , Otero M . Inflammation in osteoarthritis. Curr Opin Rheumatol. 2011;23:471–478. 21788902 10.1097/BOR.0b013e328349c2b1 PMC3937875

[67] Hwang HS , Kim HA . Chondrocyte Apoptosis in the Pathogenesis of Osteoarthritis. Int J Mol Sci. 2015;16:26035–26054. 26528972 10.3390/ijms161125943 PMC4661802

[68] Walsh DA , McWilliams DF , Turley MJ , et al. Angiogenesis and nerve growth factor at the osteochondral junction in rheumatoid arthritis and osteoarthritis. Rheumatology (Oxford). 2010;49:1852–1861. 20581375 10.1093/rheumatology/keq188 PMC2936950

[69] Xing D , Liang JQ , Li Y , et al. Identification of long noncoding RNA associated with osteoarthritis in humans. Orthop Surg. 2014;6:288–293. 25430712 10.1111/os.12147 PMC6583210

[70] Fu M , Huang G , Zhang Z , et al. Expression profile of long noncoding RNAs in cartilage from knee osteoarthritis patients. Osteoarthritis Cartilage. 2015;23:423–432. 25524778 10.1016/j.joca.2014.12.001

[71] Liu Q , Zhang X , Dai L , et al. Long noncoding RNA related to cartilage injury promotes chondrocyte extracellular matrix degradation in osteoarthritis. Arthritis Rheumatol. 2014;66:969–978. 24757148 10.1002/art.38309

[72] Henrotin Y , Sanchez C , Bay‐Jensen AC , Mobasheri A . Osteoarthritis biomarkers derived from cartilage extracellular matrix: Current status and future perspectives. Ann Phys Rehabil Med. 2016;59:145–148. 27134044 10.1016/j.rehab.2016.03.004

[73] Blanco FJ , Guitian R , Vazquez‐Martul E , de Toro FJ , Galdo F . Osteoarthritis chondrocytes die by apoptosis. A possible pathway for osteoarthritis pathology. Arthritis Rheum. 1998;41:284–289. 9485086 10.1002/1529-0131(199802)41:2<284::AID-ART12>3.0.CO;2-T

[74] Nagase H , Kashiwagi M . Aggrecanases and cartilage matrix degradation. Arthritis Res Ther. 2003;5:94–103. 12718749 10.1186/ar630 PMC165039

[75] Manabe S , Gu Z , Lipton SA . Activation of matrix metalloproteinase‐9 via neuronal nitric oxide synthase contributes to NMDA‐induced retinal ganglion cell death. Invest Ophthalmol Vis Sci. 2005;46:4747–4753. 16303975 10.1167/iovs.05-0128

[76] Glasson SS , Askew R , Sheppard B , et al. Deletion of active ADAMTS5 prevents cartilage degradation in a murine model of osteoarthritis. Nature. 2005;434:644–648. 15800624 10.1038/nature03369

[77] Rinn JL , Kertesz M , Wang JK , et al. Functional demarcation of active and silent chromatin domains in human HOX loci by noncoding RNAs. Cell. 2007;129:1311–1323. 17604720 10.1016/j.cell.2007.05.022 PMC2084369

[78] Hajjari M , Salavaty A . HOTAIR: an oncogenic long non‐coding RNA in different cancers. Cancer Biol Med. 2015;12:1–9. 25859406 10.7497/j.issn.2095-3941.2015.0006 PMC4383848

[79] Gao JZ , Li J , Du JL , Li XL . Long non‐coding RNA HOTAIR is a marker for hepatocellular carcinoma progression and tumor recurrence. Oncol Lett. 2016;11:1791–1798. 26998078 10.3892/ol.2016.4130 PMC4774541

[80] Liu FT , Qiu C , Luo HL , et al. The association of HOTAIR expression with clinicopathological features and prognosis in gastric cancer patients. Panminerva Med. 2016;58:167–174. 26964077

[81] Liu XH , Liu ZL , Sun M , Liu J , Wang ZX , De W . The long non‐coding RNA HOTAIR indicates a poor prognosis and promotes metastasis in non‐small cell lung cancer. BMC Cancer. 2013;13:464. 24103700 10.1186/1471-2407-13-464 PMC3851855

[82] Hao S , Shao Z . HOTAIR is upregulated in acute myeloid leukemia and that indicates a poor prognosis. Int J Clin Exp Pathol. 2015;8:7223–7228. 26261618 PMC4525952

[83] Kim HJ , Lee DW , Yim GW , et al. Long non‐coding RNA HOTAIR is associated with human cervical cancer progression. Int J Oncol. 2015;46:521–530. 25405331 10.3892/ijo.2014.2758 PMC4277242

[84] Zhang C , Wang P , Jiang P , et al. Upregulation of lncRNA HOTAIR contributes to IL‐1beta‐induced MMP overexpression and chondrocytes apoptosis in temporomandibular joint osteoarthritis. Gene. 2016;586:248–253. 27063559 10.1016/j.gene.2016.04.016

[85] Pachnis V , Belayew A , Tilghman SM . Locus unlinked to alpha‐fetoprotein under the control of the murine raf and Rif genes. Proc Natl Acad Sci USA. 1984;81:5523–5527. 6206499 10.1073/pnas.81.17.5523 PMC391738

[86] Matouk IJ , Halle D , Gilon M , Hochberg A . The non‐coding RNAs of the H19‐IGF2 imprinted loci: a focus on biological roles and therapeutic potential in Lung Cancer. J Transl Med. 2015;13:113. 25884481 10.1186/s12967-015-0467-3 PMC4397711

[87] Steck E , Boeuf S , Gabler J , et al. Regulation of H19 and its encoded microRNA‐675 in osteoarthritis and under anabolic and catabolic in vitro conditions. J Mol Med (Berl). 2012;90:1185–1195. 22527881 10.1007/s00109-012-0895-y

[88] Cai X , Cullen BR . The imprinted H19 noncoding RNA is a primary microRNA precursor. RNA. 2007;13:313–316. 17237358 10.1261/rna.351707 PMC1800509

[89] Dudek KA , Lafont JE , Martinez‐Sanchez A , Murphy CL . Type II collagen expression is regulated by tissue‐specific miR‐675 in human articular chondrocytes. J Biol Chem. 2010;285:24381–24387. 20529846 10.1074/jbc.M110.111328 PMC2915673

[90] Li Z , Zhao X , Zhou Y , et al. The long non‐coding RNA HOTTIP promotes progression and gemcitabine resistance by regulating HOXA13 in pancreatic cancer. J Transl Med. 2015;13:84. 25889214 10.1186/s12967-015-0442-z PMC4372045

[91] Zhang SR , Yang JK , Xie JK , Zhao LC . Long noncoding RNA HOTTIP contributes to the progression of prostate cancer by regulating HOXA13. Cell Mol Biol (Noisy‐le‐grand). 2016;62:84–88. 27064878

[92] Hsieh‐Li HM , Witte DP , Weinstein M , et al. Hoxa 11 structure, extensive antisense transcription, and function in male and female fertility. Development. 1995;121:1373–1385. 7789268 10.1242/dev.121.5.1373

[93] Yokouchi Y , Nakazato S , Yamamoto M , et al. Misexpression of Hoxa‐13 induces cartilage homeotic transformation and changes cell adhesiveness in chick limb buds. Genes Dev. 1995;9:2509–2522. 7590231 10.1101/gad.9.20.2509

[94] Kim D , Song J , Han J , Kim Y , Chun CH , Jin EJ . Two non‐coding RNAs, MicroRNA‐101 and HOTTIP contribute cartilage integrity by epigenetic and homeotic regulation of integrin‐alpha1. Cell Signal. 2013;25:2878–2887. 24018042 10.1016/j.cellsig.2013.08.034

[95] Zemmyo M , Meharra EJ , Kuhn K , Creighton‐Achermann L , Lotz M . Accelerated, aging‐dependent development of osteoarthritis in alpha1 integrin‐deficient mice. Arthritis Rheum. 2003;48:2873–2880. 14558093 10.1002/art.11246

[96] Schneider C , King RM , Philipson L . Genes specifically expressed at growth arrest of mammalian cells. Cell. 1988;54:787–793. 3409319 10.1016/s0092-8674(88)91065-3

[97] Smith CM , Steitz JA . Classification of gas5 as a multi‐small‐nucleolar‐RNA (snoRNA) host gene and a member of the 5’‐terminal oligopyrimidine gene family reveals common features of snoRNA host genes. Mol Cell Biol. 1998;18:6897–6909. 9819378 10.1128/mcb.18.12.6897 PMC109273

[98] Chen L , Yang H , Xiao Y , et al. Lentiviral‐mediated overexpression of long non‐coding RNA GAS5 reduces invasion by mediating MMP2 expression and activity in human melanoma cells. Int J Oncol. 2016;48:1509–1518. 26846479 10.3892/ijo.2016.3377

[99] Song J , Ahn C , Chun CH , Jin EJ . A long non‐coding RNA, GAS5, plays a critical role in the regulation of miR‐21 during osteoarthritis. J Orthop Res. 2014;32:1628–1635. 25196583 10.1002/jor.22718

[100] Saberi Hosnijeh F , Siebuhr AS , Uitterlinden AG , et al. Association between biomarkers of tissue inflammation and progression of osteoarthritis: evidence from the Rotterdam study cohort. Arthritis Res Ther. 2015;18:81. 10.1186/s13075-016-0976-3 PMC4818486 27039382

[101] Rausch‐Derra LC , Rhodes L , Freshwater L , Hawks R . Pharmacokinetic comparison of oral tablet and suspension formulations of grapiprant, a novel therapeutic for the pain and inflammation of osteoarthritis in dogs. J Vet Pharmacol Ther. 2016; doi: 10.1111/jvp.12306. [Epub ahead of print]. 27027634

[102] Osorio J . Osteoarthritis: Galectin‐1 damages cartilage via inflammation. Nat Rev Rheumatol. 2016;12:132–133. 10.1038/nrrheum.2016.12 26841688

[103] Kapoor M , Martel‐Pelletier J , Lajeunesse D , Pelletier JP , Fahmi H . Role of proinflammatory cytokines in the pathophysiology of osteoarthritis. Nat Rev Rheumatol. 2011;7:33–42. 21119608 10.1038/nrrheum.2010.196

[104] Brown KK , Heitmeyer SA , Hookfin EB , et al. P38 MAP kinase inhibitors as potential therapeutics for the treatment of joint degeneration and pain associated with osteoarthritis. J Inflamm (Lond). 2008;5:22. 19055838 10.1186/1476-9255-5-22 PMC2612656

[105] Pearson MJ , Jones SW . Long non‐coding RNAs in the regulation of inflammatory pathways in rheumatoid arthritis and osteoarthritis. Arthritis Rheumatol. 2016; doi: 10.1002/art.39759. [Epub ahead of print]. PMC5347907 27214788

[106] Pearson MJ , Philp AM , Heward JA , et al. Long Intergenic Noncoding RNAs Mediate the Human Chondrocyte Inflammatory Response and Are Differentially Expressed in Osteoarthritis Cartilage. Arthritis Rheumatol. 2016;68:845–856. 27023358 10.1002/art.39520 PMC4950001

[107] Lin L , Baehrecke EH . Autophagy, cell death, and cancer. Mol Cell Oncol. 2015;2:e985913. 27308466 10.4161/23723556.2014.985913 PMC4905302

[108] Szklarczyk R , Nooteboom M , Osiewacz HD . Control of mitochondrial integrity in ageing and disease. Philos Trans R Soc Lond B Biol Sci. 2014;369:20130439. 24864310 10.1098/rstb.2013.0439 PMC4032516

[109] Vanden Berghe T , Kaiser WJ , Bertrand MJ , Vandenabeele P . Molecular crosstalk between apoptosis, necroptosis, and survival signaling. Mol Cell Oncol. 2015;2:e975093. 27308513 10.4161/23723556.2014.975093 PMC4905361

[110] Zamli Z , Sharif M . Chondrocyte apoptosis: a cause or consequence of osteoarthritis? Int J Rheum Dis. 2011;14:159–166. 21518315 10.1111/j.1756-185X.2011.01618.x

[111] Pickard MR , Williams GT . The hormone response element mimic sequence of GAS5 lncRNA is sufficient to induce apoptosis in breast cancer cells. Oncotarget. 2016;7:10104–10116. 26862727 10.18632/oncotarget.7173 PMC4891107

[112] Chang L , Li C , Lan T , et al. Decreased expression of long non‐coding RNA GAS5 indicates a poor prognosis and promotes cell proliferation and invasion in hepatocellular carcinoma by regulating vimentin. Mol Med Rep. 2016;13:1541–1550. 26707238 10.3892/mmr.2015.4716 PMC4732840

[113] Gao J , Liu M , Zou Y , et al. Long non‐coding RNA growth arrest‐specific transcript 5 is involved in ovarian cancer cell apoptosis through the mitochondria‐mediated apoptosis pathway. Oncol Rep. 2015;34:3212–3221. 26503132 10.3892/or.2015.4318

[114] Yacqub‐Usman K , Pickard MR , Williams GT . Reciprocal regulation of GAS5 lncRNA levels and mTOR inhibitor action in prostate cancer cells. Prostate. 2015;75:693–705. 25650269 10.1002/pros.22952

[115] Doherty M . Synovial inflammation and osteoarthritis progression: effects of nonsteroidal antiinflammatory drugs. Osteoarthritis Cartilage. 1999;7:319–320. 10329313 10.1053/joca.1998.0179

[116] Lambert C , Dubuc JE , Montell E , et al. Gene expression pattern of cells from inflamed and normal areas of osteoarthritis synovial membrane. Arthritis Rheumatol. 2014;66:960–968. 24757147 10.1002/art.38315 PMC4033569

[117] Kang Y , Song J , Kim D , et al. PCGEM1 stimulates proliferation of osteoarthritic synoviocytes by acting as a sponge for miR‐770. J Orthop Res. 2016;34:412–418. 26340084 10.1002/jor.23046

[118] Mapp PI , Walsh DA . Mechanisms and targets of angiogenesis and nerve growth in osteoarthritis. Nat Rev Rheumatol. 2012;8:390–398. 22641138 10.1038/nrrheum.2012.80

[119] Merry P , Grootveld M , Blake DR . Hypoxic‐reperfusion injury in inflamed joints. Lancet. 1989;1:1023. 10.1016/s0140-6736(89)92668-8 2565501

[120] O'Hara BP , Urban JP , Maroudas A . Influence of cyclic loading on the nutrition of articular cartilage. Ann Rheum Dis. 1990;49:536–539. 2383080 10.1136/ard.49.7.536 PMC1004145

[121] Hamilton JL , Nagao M , Levine BR , Chen D , Olsen BR , Im HJ . Targeting VEGF and Its Receptors for the Treatment of Osteoarthritis and Associated Pain. J Bone Miner Res. 2016;31:911–924. 27163679 10.1002/jbmr.2828 PMC4863467

[122] Walsh DA , Bonnet CS , Turner EL , Wilson D , Situ M , McWilliams DF . Angiogenesis in the synovium and at the osteochondral junction in osteoarthritis. Osteoarthritis Cartilage. 2007;15:743–751. 17376709 10.1016/j.joca.2007.01.020

[123] Bonnet CS , Walsh DA . Osteoarthritis, angiogenesis and inflammation. Rheumatology (Oxford). 2005;44:7–16. 15292527 10.1093/rheumatology/keh344

[124] Carmeliet P , Jain RK . Angiogenesis in cancer and other diseases. Nature. 2000;407:249–257. 11001068 10.1038/35025220

[125] Zhou Y , Zhang X , Klibanski A . MEG3 noncoding RNA: a tumor suppressor. J Mol Endocrinol. 2012;48:R45–R53. 22393162 10.1530/JME-12-0008 PMC3738193

[126] Veikkola T , Alitalo K . VEGFs, receptors and angiogenesis. Semin Cancer Biol. 1999;9:211–220. 10343072 10.1006/scbi.1998.0091

[127] Su W , Xie W , Shang Q , Su B . The Long Noncoding RNA MEG3 Is Downregulated and Inversely Associated with VEGF Levels in Osteoarthritis. Biomed Res Int. 2015;2015:356893. 26090403 10.1155/2015/356893 PMC4454735

[128] Pal S , Datta K , Mukhopadhyay D . Central role of p53 on regulation of vascular permeability factor/vascular endothelial growth factor (VPF/VEGF) expression in mammary carcinoma. Cancer Res. 2001;61:6952–6957. 11559575

[129] Zheng ZM , Tang S , Tao M . Development of resistance to RNAi in mammalian cells. Ann N Y Acad Sci. 2005;1058:105–118. 16394130 10.1196/annals.1359.019 PMC1462965

[130] Ichim TE , Li M , Qian H , et al. RNA interference: a potent tool for gene‐specific therapeutics. Am J Transplant. 2004;4:1227–1236. 15268723 10.1111/j.1600-6143.2004.00530.x PMC7175948

[131] Svoboda P . Off‐targeting and other non‐specific effects of RNAi experiments in mammalian cells. Curr Opin Mol Ther. 2007;9:248–257. 17608023

[132] Gerard X , Garanto A , Rozet JM , Collin RW . Antisense Oligonucleotide Therapy for Inherited Retinal Dystrophies. Adv Exp Med Biol. 2016;854:517–524. 26427454 10.1007/978-3-319-17121-0_69

[133] Bennett CF , Swayze EE . RNA targeting therapeutics: molecular mechanisms of antisense oligonucleotides as a therapeutic platform. Annu Rev Pharmacol Toxicol. 2010;50:259–293. 20055705 10.1146/annurev.pharmtox.010909.105654

[134] Zhou T , Kim Y , MacLeod AR . Targeting Long Noncoding RNA with Antisense Oligonucleotide Technology as Cancer Therapeutics. Methods Mol Biol. 2016;1402:199–213. 26721493 10.1007/978-1-4939-3378-5_16

[135] Gutschner T , Hammerle M , Eissmann M , et al. The noncoding RNA MALAT1 is a critical regulator of the metastasis phenotype of lung cancer cells. Cancer Res. 2013;73:1180–1189. 23243023 10.1158/0008-5472.CAN-12-2850 PMC3589741

[136] Grunweller A , Hartmann RK . Locked nucleic acid oligonucleotides: the next generation of antisense agents? BioDrugs. 2016;21:235–243. 10.2165/00063030-200721040-00004 17628121

[137] Michalik KM , You X , Manavski Y , et al. Long noncoding RNA MALAT1 regulates endothelial cell function and vessel growth. Circ Res. 2014;114:1389–1397. 24602777 10.1161/CIRCRESAHA.114.303265

[138] Pastori C , Kapranov P , Penas C , et al. The Bromodomain protein BRD4 controls HOTAIR, a long noncoding RNA essential for glioblastoma proliferation. Proc Natl Acad Sci USA. 2015;112:8326–8331. 26111795 10.1073/pnas.1424220112 PMC4500283

[139] Tsai MC , Spitale RC , Chang HY . Long intergenic noncoding RNAs: new links in cancer progression. Cancer Res. 2011;71:3–7. 21199792 10.1158/0008-5472.CAN-10-2483 PMC3057914

[140] Kim YG , Cha J , Chandrasegaran S . Hybrid restriction enzymes: zinc finger fusions to Fok I cleavage domain. Proc Natl Acad Sci USA. 1996;93:1156–1160. 8577732 10.1073/pnas.93.3.1156 PMC40048

[141] Gutschner T , Baas M , Diederichs S . Noncoding RNA gene silencing through genomic integration of RNA destabilizing elements using zinc finger nucleases. Genome Res. 2011;21:1944–1954. 21844124 10.1101/gr.122358.111 PMC3205578

[142] Nugent M . MicroRNAs: exploring new horizons in osteoarthritis. Osteoarthritis Cartilage. 2016;24:573–580. 26576510 10.1016/j.joca.2015.10.018

[143] Portal‐Nunez S , Esbrit P , Alcaraz MJ , Largo R . Oxidative stress, autophagy, epigenetic changes and regulation by miRNAs as potential therapeutic targets in osteoarthritis. Biochem Pharmacol. 2016;108:1–10. 26711691 10.1016/j.bcp.2015.12.012

[144] Vicente R , Noel D , Pers YM , Apparailly F , Jorgensen C . Deregulation and therapeutic potential of microRNAs in arthritic diseases. Nat Rev Rheumatol. 2016;12:211–220. 26698025 10.1038/nrrheum.2015.162

[145] Fekrazad R , Naghdi N , Nokhbatolfoghahaei H , Bagheri H . The combination of laser therapy and metal nanoparticles in cancer treatment originated from epithelial tissues: a literature review. J Lasers Med Sci. 2016;7:62–75. 27330701 10.15171/jlms.2016.13 PMC4909014

[146] Zhao K , Li D , Shi C , et al. Biodegradable polymeric nanoparticles as the delivery carrier for drug. Curr Drug Deliv. 2016;13:494–499. 27230997 10.2174/156720181304160521004609

[147] Fang K , Liu P , Dong S , et al. Magnetofection based on superparamagnetic iron oxide nanoparticle‐mediated low lncRNA HOTAIR expression decreases the proliferation and invasion of glioma stem cells. Int J Oncol. 2016;49:509–518. 27277755 10.3892/ijo.2016.3571 PMC4922836

